# *Arabidopsis* GEX1 Is a Nuclear Membrane Protein of Gametes Required for Nuclear Fusion During Reproduction

**DOI:** 10.3389/fpls.2020.548032

**Published:** 2020-10-12

**Authors:** Shuh-ichi Nishikawa, Yuki Yamaguchi, Chiharu Suzuki, Ayaka Yabe, Yuzuru Sato, Daisuke Kurihara, Yoshikatsu Sato, Daichi Susaki, Tetsuya Higashiyama, Daisuke Maruyama

**Affiliations:** ^1^Biology Program, Faculty of Science, Niigata University, Niigata, Japan; ^2^Graduate School of Science and Technology, Niigata University, Niigata, Japan; ^3^Japan Science and Technology Agency, PRESTO, Nagoya, Japan; ^4^Institute of Transformative Bio-Molecules, Nagoya University, Nagoya, Japan; ^5^Division of Biological Science, Graduate School of Science, Nagoya University, Nagoya, Japan; ^6^Kihara Institute for Biological Research, Yokohama City University, Yokohama, Japan; ^7^Department of Biological Sciences, Graduate School of Science, University of Tokyo, Tokyo, Japan

**Keywords:** nuclear fusion, female gametogenesis, fertilization, membrane fusion, *Arabidopsis thaliana*

## Abstract

During the life cycle of flowering plants, nuclear fusion, or karyogamy, occurs three times: once during female gametogenesis, when the two polar nuclei fuse in the central cell, and twice during double fertilization. In *Arabidopsis thaliana*, nuclear fusion events during sexual reproduction proceed without the breakdown of the nuclear envelope, indicating that nuclear membrane fusion is essential for the completion of this process. *Arabidopsis* gamete expressed 1 (GEX1) is a membrane protein that is conserved among plant species. GEX1 shares homology with the yeast karyogamy protein Kar5, which is primarily expressed in the nuclear membrane. The GEX1 family represents a putative karyogamy factor. Herein, we show that GEX1 is required for the nuclear fusion events in *Arabidopsis* reproduction. GEX1-deficient mature female gametophytes were found to contain two unfused polar nuclei in close proximity within the central cell. Electron microscopy showed that the outer membrane of the polar nuclei was connected via the endoplasmic reticulum, whereas the inner membrane remained unfused. These results indicate that GEX1 is involved in polar nuclear membrane fusion following the fusion of the outer nuclear membrane. Furthermore, sperm nuclear fusion events were defective in the fertilized egg and central cell following plasmogamy in the fertilization of *gex1-1* female gametophytes by *gex1-1* pollen. An analysis of GEX1 localization in the female gametophyte using a transgenic line expressing GFP-tagged GEX1 driven by the *GEX1* promoter showed that GEX1 is a nuclear membrane protein in the egg and central cell. Time-lapse live-cell imaging showed that in developing female gametophytes, the nuclear GFP-GEX1 signal was first detectable in the central cell shortly before the polar nuclei came in close contact, and then in the egg cell. Thus, we suggest that the GEX1-family proteins are nuclear membrane proteins involved in karyogamy in the reproduction of eukaryotes including flowering plants.

## Introduction

Nuclear fusion, or karyogamy, is essential for the sexual reproduction of various organisms including plants, animals, and fungi. During the life cycle of flowering plants, nuclear fusion occurs three times; two of these events are sperm nuclear fusion during double fertilization. Two sperm cells released from a pollen tube fertilize the egg and central cell, giving rise to the embryo and endosperm, respectively. After the fusion of a sperm cell with the egg or central cell, the sperm nucleus fuses with the nucleus of a female gamete (Dresselhaus et al., 2016). The third nuclear fusion occurs during the development of female gametophytes (Yadegari and Drews, 2004; Drews and Koltunow, 2011), which develop in the ovule. The developmental pattern of most flowering plants, including *Arabidopsis thaliana*, is the *Polygonum* type. A single megaspore produced by meiosis undergoes three rounds of mitosis. After the second mitosis, a four-nucleate female gametophyte is produced. Cellularization starts after the third mitosis, resulting in the formation of a seven-celled female gametophyte, which consists of 1 egg cell, 1 central cell, 2 synergid cells, and 3 antipodal cells. The central cell contains two polar nuclei that migrate toward the center of the female gametophyte. In *Arabidopsis* and other species, the polar nuclei fuse before pollination to form the secondary nucleus (Yadegari and Drews, 2004; Drews and Koltunow, 2011).

Previously, we showed that the immunoglobulin binding protein (BiP), a molecular chaperone of the heat shock protein 70 (Hsp70) family in the endoplasmic reticulum (ER), is involved in the fusion of polar nuclei with its regulatory partners, ER-resident J-domain containing proteins (J-proteins). The *Arabidopsis* genome harbors three *BIP* genes (*BIP1-BIP3*) with redundant functions (Maruyama et al., 2014a, 2015). Female gametophytes of the *bip1 bip2* double mutant, which lacks two ubiquitously expressed *BIP* genes, contain two unfused polar nuclei in close proximity (Maruyama et al., 2010). A mutant analysis revealed the involvement of three luminal ER-resident J-proteins, ERdj3A, ERdj3B, and P58^IPK^ (Yamamoto et al., 2008), in the polar nuclear fusion process; P58^IPK^/ERdj3A and P58^IPK^/ERdj3B function in the fusion of outer and inner nuclear membranes of polar nuclei, respectively, as partners for BiP (Maruyama et al., 2014b). We also showed that BiP and ER-resident J-proteins are involved in sperm nuclear fusion after plasmogamy (Maruyama et al., 2020). BiP and ER-resident J-proteins probably regulate the functions of nuclear membrane proteins to promote nuclear fusion during *Arabidopsis* reproduction.

The GEX1/Kar5 family proteins have been identified as key factors for nuclear fusion. Budding yeast Kar5 is an integral nuclear membrane protein that is essential for nuclear fusion during yeast mating (Beh et al., 1997). Zebrafish Brambleberry (Bmb) protein, which shares sequence identity with yeast Kar5, is a nuclear membrane protein that is essential for pronucleus fusion in the zygote and for karyomere fusion during early embryogenesis (Abrams et al., 2012). The gamete expressed 1 (GEX1) proteins in plants and protists are functional orthologs of Kar5 (Speijer et al., 2015). The GEX1 orthologs of *Chlamydomonas* and *Plasmodium* are nuclear membrane proteins required for sexual reproduction (Ning et al., 2013). Although Kar5, Bmb, and GEX1 differ widely in size and degree of sequence identity, they all contain a well-defined Cys-rich domain (CRD) within their N-terminal region (Ning et al., 2013). The GEX1 orthologs are predicted to possess three transmembrane domains in their C-terminal region, in contrast to Kar5 and Bmb, which possess two transmembrane domains. In *Arabidopsis*, GEX1 was identified by screening genes that are expressed in the sperm cells of pollen (Engel et al., 2005). GEX1 is expressed in female gametophytes, and the roles for GEX1 in gametophyte development and early embryogenesis have been suggested (Alandete-Saez et al., 2011).

Here, we analyzed the roles of GEX1 in nuclear fusion during *Arabidopsis* reproduction. The results showed that female gametophytes containing the *gex1* mutation are defective in the fusion of polar nuclei. Sperm nuclear fusion was defective in both fertilized egg and central cells after the fertilization of *gex1* mutant female gametophytes by *gex1* mutant pollen. Using a green fluorescent protein (GFP)-fusion protein, we show that GEX1 is a nuclear membrane protein expressed specifically in the egg and central cells in female gametophytes. These results indicate that GEX1 is a conserved nuclear membrane protein that is essential for nuclear fusion in the reproduction of flowering plants.

## Materials and Methods

### Plant Materials and Growth Conditions

*Arabidopsis thaliana* ecotype Columbia (Col), Wassilewskija (Ws), and *qrt1-2* (CS8846; Col-3) were used as the wild type. The *gex1-1* (CS817261; Col-3) and *gex1-2* (FLAG_484E09; Ws) mutant alleles have been described by Alandete-Saez et al. (2011). The seeds were surface sterilized and sown on soil or Murashige and Skoog (MS) medium (Fuji Film Wako, Osaka, Japan) containing 0.7% agar and 1% sucrose. The plants were grown at 22°C under continuous light.

### Plasmid Construction and Plant Transformation

Primers used for plasmid construction are listed in Supplementary Table 1. The total RNA was extracted from the flowers of *A. thaliana* Col plants using the RNeasy Plant Mini Kit (Qiagen, Tokyo, Japan) and reverse transcribed to cDNA using the SuperScriptIII first-strand synthesis system (Thermo Fisher Scientific K.K., Tokyo, Japan) according to the manufacturer’s protocol. A cDNA fragment for *GEX1* was PCR-amplified using primer set GEX1-YY2/GEX1-YY5 and first-strand cDNA as a template. The amplified 1.8 kb DNA fragment was cloned into the pENTR/D-TOPO vector to generate pSNA134. The *GFP-GEX1* fusion gene was constructed as follows. A DNA fragment corresponding to the signal sequence (amino acid residues 1–27) of GEX1 was PCR amplified from the *GEX1* cDNA using primers GFPGEX1-1 and GFPGEX1-2. A DNA fragment corresponding to amino acid residues 1–239 of the synthetic green fluorescent protein (GFP) was amplified from pSK35SGFP (Yamamoto et al., 2010) using primers GFPGEX1-3 and GFPGEX1-4. A DNA fragment corresponding to amino acid residues 28–593 of Gex1 with a stop codon was PCR amplified from the *GEX1* cDNA using primers GFPGEX1-5 and GFPGEX1-6. All three amplified DNA fragments were ligated using the In-Fusion HD Cloning Kit (Takara Bio Inc., Kusatsu, Japan), and then cloned into the pENTR/D-TOPO vector, to generate pSNA133. A DNA fragment corresponding to 1.6 kb of the region upstream of the start codon of *GEX1* was amplified from *A. thaliana* (Col) genomic DNA by PCR using primer set GEX1proF/GEX1proR. This was then cloned into the *Hin*dIII site of pGWB501 (Nakagawa et al., 2007) using the In-Fusion HD Cloning Kit to generate pSNA136. The *GEX1* and *GFP-GEX1* constructs from pSNA134 and pSNA133, respectively, were introduced into pSNA136 using LR clonase II (Thermo Fisher Scientific K.K., Tokyo, Japan). Plasmids used to express HISTONE H2B-GFP or HISTONE H2B-tandem tomato (tdTomato) from the *RPS5A* promoter have been described previously (Maruyama et al., 2013).

To generate transgenic lines, *Agrobacterium tumefaciens* strain GV3101 was introduced into *Arabidopsis* plants using the floral-dip method (Clough and Bent, 1998). The transgenic plants were selected on MS agar plates containing 50 μg/mL hygromycin or 50 μg/mL kanamycin, and subsequently transferred to soil.

### Microscopy

The ovules were prepared for confocal laser-scanning microscopy (CLSM) as described by Christensen et al. (1997). To observe GFP and tdTomato fluorescence, the ovules were dissected from the pistils and mounted in a multi-well glass bottom dish (D141400; Matsunami Glass, Osaka, Japan) containing 400 μL of ovule culture medium (Gooh et al., 2015). To analyze sperm nuclear fusion, flowers of the *gex1-1*/+ line expressing HISTONE H2B-tdTomato driven by the *RPS5A* promoter were emasculated at late stage 12 (Smyth et al., 1990) and pollinated with pollen from the *gex1-1*/+ line expressing HISTONE H2B-GFP under the *RPS5A* promoter. Seven-hours after pollination, the ovules were dissected from the pistils and mounted in a multi-well glass bottom dish containing 400 μL of ovule culture medium. For the quantitative analysis, the pistils were fixed 8 h after pollination and cleared by ClearSee (Fuji Film Wako, Osaka, Japan) as described previously (Kurihara et al., 2015).

A Leica TCS-SP8 confocal microscope fitted with a 20 × multi-immersion objective lens (PL APO CS2 20×/0.75 IMM CORR HC; Leica Microsystems), 40 × water-immersion objective lens (PL APO CS2 40×/1.10 W CORR HCX; Leica Microsystems), 63 × water-immersion objective lens (HC PL APO 63×/1.20 W CORR CS2; Leica Microsystems), or 63 × glycerol-immersion objective lens (HC PL APO 63×/1.30 GLYC CORR CS2; Leica Microsystems, Tokyo, Japan) was used for CLSM. For GFP fluorescence, images were captured at 495–540 nm after excitation at 488 nm with solid-state laser. For tdTomato fluorescence, images were captured at 560–650 nm after excitation at 552 nm with solid-state laser. For autofluorescence, images were captured at 490–778 nm after excitation at 484 nm using white light laser. For GFP and tdTomato fluorescence, images were captured at 493–549 nm and at 559–650 nm after excitation at 488 nm and 554 nm, respectively, using white light laser. To create maximum intensity projection images, the acquired images were processed using LASX software (Leica Microsystems, Tokyo, Japan).

Developing seeds in siliques were fixed in 10% (v/v) acetic acid and 90% (v/v) ethanol for 10 min, and then rehydrated in an ethanol series (80, 60, and 40% ethanol (v/v) for 10 min each). The samples were cleared in 70% (w/w) chloral hydrate and 8.8% (w/w) glycerol. The cleared seeds were dissected from the siliques and observed using Axio IMAGER M1 (Zeiss, Oberkochen, Germany).

### Time-Lapse Live-Cell Imaging

Live-cell imaging of developing ovules was performed as previously described (Susaki et al., 2020). The ovules were dissected from stage 12 flowers of wild-type plants expressing GFP-GEX1 driven by the *GEX1* promoter, and HISTONE H2B-tdTomato driven by the *RPS5A* promoter, and then mounted in a multi-well glass bottom dish containing 400 μL of ovule culture medium. Time-lapse confocal imaging was performed using an inverted microscope (IX-83; Olympus, Tokyo, Japan) equipped with a disk-scan confocal system (CSU-W1, Yokogawa Electric, Tokyo, Japan), 488 and 552 nm LD lasers (Sapphire; Coherent Japan, Tokyo, Japan), and an EM-CCD camera (iXon3, Andor Technology, Belfast, United Kingdom). Time-lapse images were acquired every 5 min with 21 *z*-planes at 1 μm intervals, using a silicone-immersion objective lens (UPLSAPO60XS2; Olympus, Tokyo, Japan). The images were analyzed using MetaMorph Version 7.8.10.0. (Molecular Devices Japan, Tokyo, Japan).

### Transmission Electron Microscopy

Unfertilized ovules were dissected from the pistils and fixed in 2% glutaraldehyde, 4% paraformaldehyde, and 50 mM sodium cacodylate (pH 7.4) at 4°C overnight. The tissue segments were washed in buffer and fixed in 2% aqueous osmium tetroxide for 6 h at 4°C. The tissue was then dehydrated in a graded ethanol series, transferred into propylene oxide, infiltrated, and embedded in Quetol 651. Series of thin sections (80 nm) were stained with 2% aqueous uranyl acetate and lead citrate, and examined at 80 kV under a JEOL JEM 1400 Plus electron microscope (JEOL Ltd., Tokyo, Japan). Digital images were obtained using a CCD camera (VELETA; Olympus Soft Imaging Solutions, Tokyo, Japan).

### Image Processing

Fiji (Schindelin et al., 2012) was used to edit images and time-lapse movies. All other images were processed for publication using Adobe Photoshop CC (Adobe Systems Inc., San Jose, CA, United States).

## Results

### GEX1 Mutant Female Gametophytes Are Defective in Polar Nuclear Fusion

**FIGURE 1:**
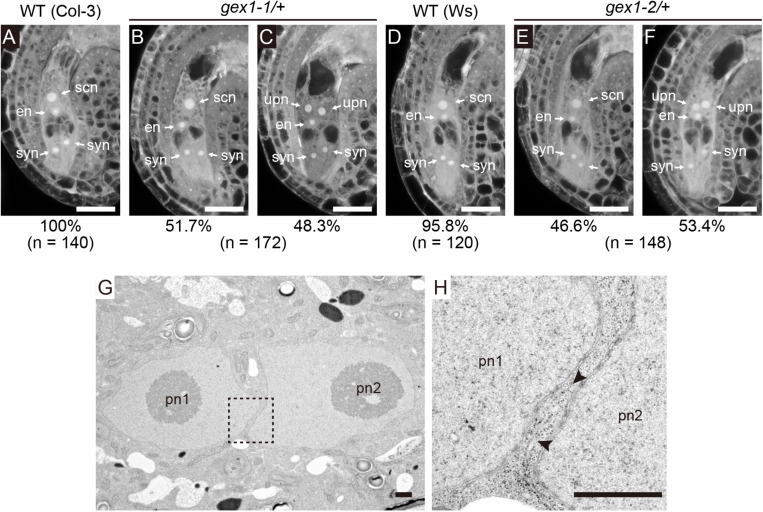
The *gex1* mutation causes defects in the fusion of polar nuclei during female gametogenesis. **(A–F)** Confocal laser-scanning microscopy (CLSM) of the ovules of the wild-type (Col-3, *qrt1-2*) **(A)**, *gex1-1*/+ **(B,C)**, wild-type (Ws) **(D)**, and *gex1-2*/+ **(E,F)** plants. Half of the ovules from the *gex1-1*/+ and *gex1-2*/+ plants appeared morphologically normal **(B,E)**, whereas the polar nuclei were unfused in the remaining ovules **(C,F)**. Percentage of ovule types observed is presented below the panel with the total number of ovules observed. syn, synergid nucleus; en, egg nucleus; scn, secondary nucleus; upn, unfused polar nucleus. Bars = 20 μm. **(G,H)** Electron micrographs of the central cell of a *gex1-1* female gametophyte containing unfused polar nuclei. The region indicated with a box in **(G)** is magnified in **(H)**. The arrowheads show membrane bridges connecting the outer nuclear membrane and the endoplasmic reticulum (ER) membrane. pn1, polar nucleus 1; pn2, polar nucleus 2. Scale bars = 1 μm.

A mutant analysis was performed to examine the roles of GEX1 in polar nuclear fusion in *A. thaliana*. We obtained mutant lines carrying one of the two *GEX1* alleles (*gex1-1* and *gex1-2*; Alandete-Saez et al., 2011). The third *GEX1* allele, *gex1-3*, has also been reported by Alandete-Saez et al. (2011), but it was not available from the stock center. An analysis of mature-stage female gametophytes from parental wild-type lines by CLSM revealed two synergid cell nuclei, an egg nucleus, and a secondary nucleus generated by polar nuclear fusion (Christensen et al., 1997) (Figures 1A,D). As the homozygous *gex1* mutant is lethal, female gametophytes from the *gex1*/+ plants were analyzed. We found that approximately 50% of female gametophytes of both *gex1-1*/+ and *gex1-2*/+ plants contained unfused polar nuclei (Figures 1B,C,E,F), and this was similar to the percentage observed in *bip1 bip2*, *erdj3a p58^*ipk*^*, and *erdj3b p58^*ipk*^*-mutant female gametophytes (Maruyama et al., 2010, 2014b). We introduced a construct containing *GEX1* cDNA driven by the *GEX1* promoter (*pGEX1:GEX1*) into the *gex1-1*/+ plants. Five independent transgenic *gex1-1*/+ lines homozygous for *pGEX1:GEX1* were isolated. More than 90% of the ovules contained a single secondary nucleus (Supplementary Table 2). These results indicate that the *gex1* mutation leads to defects in polar nuclear fusion during female gametogenesis.

We analyzed nuclear membrane fusion in the ovules of the *gex1-1* mutant by transmission electron microscopy (*n* = 4). In all ovules, two unfused polar nuclei were observed in close contact (Figure 1G and Supplementary Figures 1A,C). ER cisterns connecting the outer nuclear membranes of the unfused polar nuclei were frequently observed (Figure 1H and Supplementary Figures 1B,D arrowheads). These results indicate that *gex1-1-*mutant female gametophytes were defective in nuclear-membrane fusion following fusion of the outer nuclear membranes, and this is similar to that observed in *erdj3b p58^*ipk*^* double-mutant female gametophytes (Maruyama et al., 2014b).

### GEX1 Is a Nuclear Membrane Protein Expressed Specifically in Gametes During Female Gametogenesis

**FIGURE 2:**
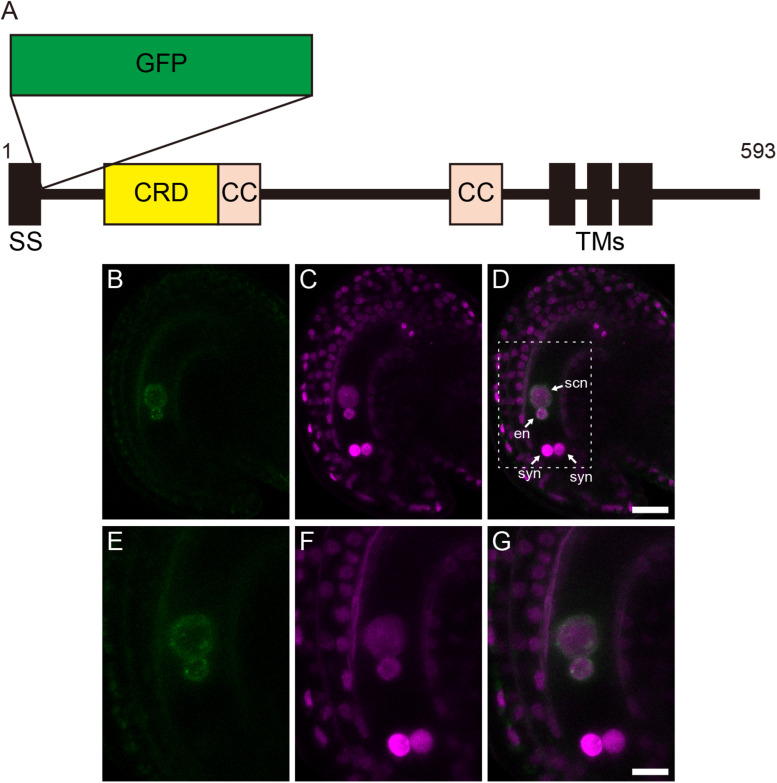
Gamete expressed 1 (GEX1) is localized to the nuclear membrane of the egg and central cells in mature female gametophytes. **(A)** Schematic representation of a green fluorescent protein (GFP)-GEX1 fusion protein. GEX1 has 1 predicted *N*-terminal signal sequence (SS), 1 Cys-rich domain (CRD), 2 coiled-coil domains (CC), and 3 transmembrane domains (TMs). In the GFP-GEX1 fusion protein that was used in this study, the GFP was inserted after the SS. **(B–G)** CLSM images of a mature wild-type ovule expressing GFP-GEX1 driven by the *GEX1* promoter and HISTONE H2B-tdTomato driven by the *RPS5A* promoter. GFP fluorescence **(B,E)**, tdTomato fluorescence **(C,F)**, and merged **(D,G)** images are shown. **(E–G)** Magnification of the boxed region in **(D)**. syn, synergid nucleus; en, egg nucleus; scn, secondary nucleus. Scale bars = 20 μm **(D)**, 10 μm **(G)**.

The localization of GEX1 in female gametophytes was analyzed by developing transgenic plants carrying *GFP*-fused *GEX1* driven by the *GEX1* promoter (*pGEX1:GFP-GEX1*). We inserted synthetic GFP (Niwa et al., 1999) into GEX1 after the putative signal sequence (between Trp-27 and Gly-28; Figure 2A). A construct that ubiquitously expresses HISTONE H2B tagged with tandem tomato (tdTomato) driven by the *RPS5A* promoter (*pRPS5A:H2B-tdTomato*) was introduced to visualize the nuclei. The CLSM analysis of a mature female gametophyte revealed GFP signals exclusively in the nuclei of the egg and central cells (Figure 2B and Supplementary Figure 2). The GFP signals were observed at the periphery of the gamete nuclei (Figure 2E), indicating that GEX1 is a nuclear membrane protein. We also observed punctate GFP signals in the nuclei, suggesting that GEX1 is not uniformly distributed in the nuclear membrane. The RNA-Seq analysis of the isolated synergid, egg, and central cells (Susaki et al., 2020) showed *GEX1* expression in the egg and central cells (Supplementary Figure 3). The expression of *GEX1* was higher in the egg cell than in the central cell.

**FIGURE 3:**
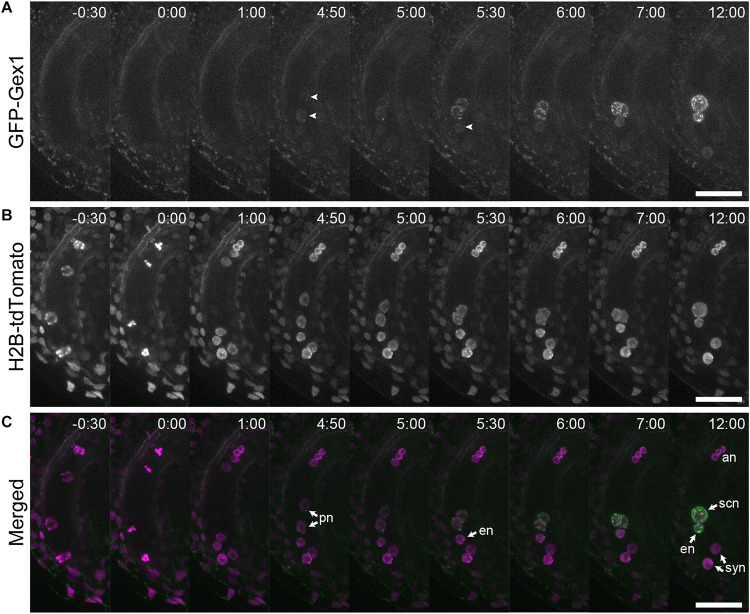
Time-lapse analysis showing the expression and localization of GFP-GEX1 during female gametophyte development. The ovules containing a developing female gametophyte expressing GFP-GEX1 driven by the *GEX1* promoter and HISTONE H2B-tdTomato driven by the *RPS5A* promoter were incubated in the ovule culture media (Gooh et al., 2015). Images were captured at 5-min intervals. Time (h:min) from the metaphase of the third mitotic division is shown in each panel. GFP fluorescence **(A)**, tdTomato fluorescence **(B)**, and merged **(C)** images are shown. syn, synergid nucleus; en, egg nucleus; scn, secondary nucleus; pn, polar nucleus; an, antipodal nucleus. The arrowheads in **(A)** denote GFP fluorescence in the central (time point 4:50) and egg cells (time point 5:30). Scale bars = 20 μm.

We analyzed the localization of GEX1 in developing female gametophytes. The ovules were isolated from stage 12 (Smyth et al., 1990) flowers of the transgenic plants and mounted in a glass-bottom dish containing ovule culture medium. Time-lapse live cell imaging was performed from the four-nucleated stage. GFP fluorescence was not observed in the four-nucleated female gametophytes or in female gametophytes following the third mitotic division (Figure 3, timepoints -0 h 30 min and 1 h 00 min; Supplementary Movie 1). GFP fluorescence was first observed in migrating polar nuclei (Figure 3A, timepoint 4 h 50 min arrowheads; Supplementary Movie 1 and 2) when the polar nuclei were in close contact (−4.4 ± 36 min from the contact of polar nuclei, *n* = 9). The GFP signals in the egg cell were observed at 81 ± 44 min (*n* = 9) following the appearance of GFP signals in the central cell (Figure 3A, timepoint 5 h 30 min arrowhead). These results indicate that in developing female gametophytes, expression driven by the *GEX1* promoter started after cellularization.

As *GEX1* has been reported to be expressed in the pollen (Engel et al., 2005; Alandete-Saez et al., 2011), we analyzed the localization of GFP-GEX1 in the pollen by CLSM. Punctate GFP signals were observed at the periphery of the vegetative nucleus (Supplementary Figure 4), indicating nuclear membrane localization of GFP-GEX1 in the pollen. The expression of GFP-GEX1 in the sperm cells was weak. We analyzed three independent *pGEX1:GFP-GEX1* transgenic lines, but we were not able to analyze the localization of GFP-GEX1 in the sperm cells.

### GEX1 Is Required for Sperm Nuclear Fusion After Fertilization

**FIGURE 4:**
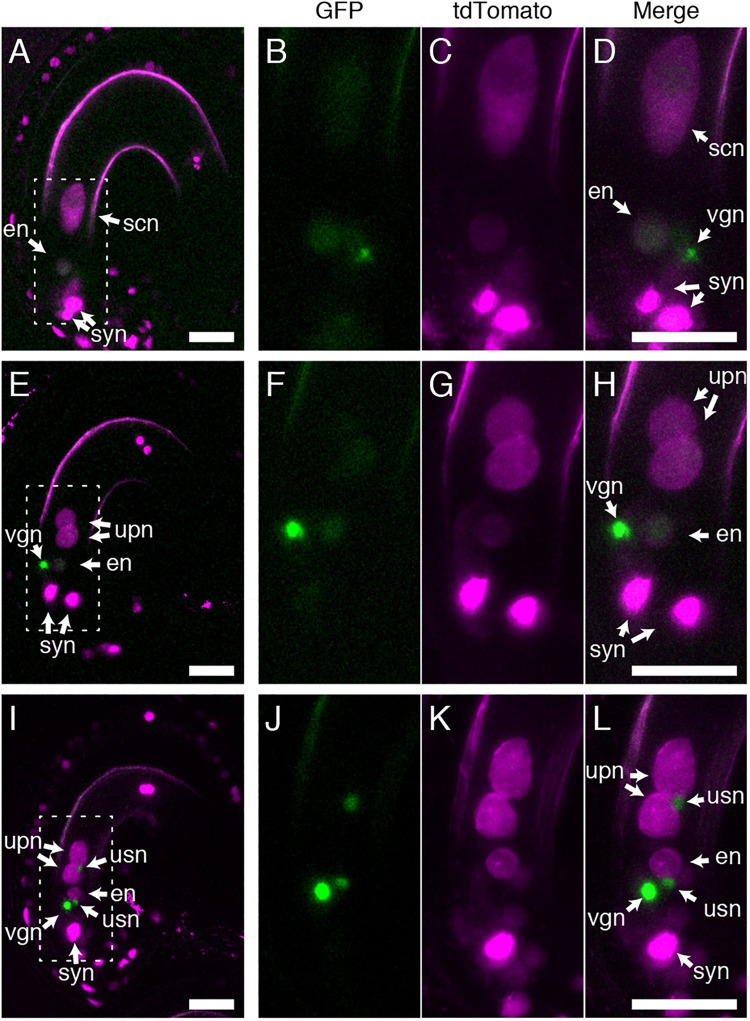
Sperm nuclear fusion was defective after the fertilization of a *gex1-1* female gametophyte by *gex1-1* pollen. The pistils of the *gex1-1*/+ line expressing HISTONE H2B-tdTomato driven by the *RPS5A* promoter were pollinated with pollen from the *gex1-1/*+ line expressing HISTONE H2B-GFP driven by the *RPS5A* promoter. Seven-hours after pollination, the ovules were dissected from the pistils and analyzed by CLSM. **(A–D)** Images of a fertilized wild-type female gametophyte. **(E–H)** Images of a fertilized *gex1-1* female gametophyte with successful sperm nuclear fusion. **(I–L)** Images of a fertilized *gex1-1* female gametophyte showing defects in sperm nuclear fusion. GFP fluorescence **(B,F,J)**, tdTomato fluorescence **(C,G,K)**, and merged **(A,D,E,H,I,L)** images are shown. **(B–D)**, **(F–H),** and **(J–L)** show the boxed regions in **(A)**, **(E)**, and **(I)**, respectively. In **(I–L)**, one of the synergid nuclei is out of focus and is not shown. syn, synergid nucleus; en, egg nucleus; scn, secondary nucleus; upn, unfused polar nucleus; usn, unfused sperm nucleus; vgn, vegetative nucleus of pollen tube. Scale bars = 20 μm.

As BiP and ER-resident J-proteins function in both polar and sperm nuclear fusion (Maruyama et al., 2020), we assessed whether GEX1 is also required for sperm nuclear fusion after fertilization. Pistils of the *gex1-1*/+ plants expressing *pRPS5A:H2B-tdTomato* were pollinated with pollen from the *gex1-1*/+ plants expressing HISTONE H2B tagged with GFP driven by the *RPS5A* promoter. The ovules were dissected from the pistils 7 h after pollination (HAP) and analyzed by CLSM. The fertilized ovules were distinguished by the presence of a vegetative nucleus of the pollen tube. Two types of fertilized female gametophytes were observed, with one or two nuclei labeled with H2B-tdTomato in the central cell, corresponding to the wild-type or *gex1-1* female gametophytes, respectively (Figure 4). In the fertilized wild-type female gametophytes, the GFP signals from the sperm nuclei were not observed probably due to diffusion within the fertilized egg and central cell nuclei (Figures 4A–D).

Two types of fertilized *gex1-1* female gametophytes containing the unfused polar nuclei in the central cell were observed. In one type, the GFP signals from the sperm nuclei were not visible (Figures 4E–H), and these were similar to the fertilized wild-type female gametophytes. In the other type, punctate GFP signals were observed adjacent to the unfused polar nuclei and egg nucleus (Figures 4I–L), indicating defective sperm nuclear fusion. We analyzed the proportion of ovules defective in sperm nuclear fusion. We pollinated the pistils of the *gex1-1*/+ or wild-type plants expressing HISTONE H2B-tdTomato with pollen from the *gex1-1*/+ or wild-type plants expressing HISTONE H2B-GFP. The pistils were fixed in 8HAP and analyzed by CLSM. We analyzed 66 fertilized *gex1-1* ovules that were defective in the fusion of polar nuclei and found that 48.5% of the ovules showed defects in sperm nuclear fusion (Table 1). Sperm nuclear fusion was defective in both egg and central cells in all cases. As the *gex1-1*/+ plants produce wild-type and *gex1-1* pollen in a 1:1 ratio, that observed was consistent with defective sperm nuclear fusion when the *gex1-1* female gametophytes were fertilized by *gex1-1* pollen. These results indicate that GEX1 is required for both polar and sperm nuclear fusion upon fertilization.

**TABLE 1:** Sperm nuclear fusion defect observed after the fertilization of the *gex1-1* female gametophytes by *gex1-1* pollen.

Genetic cross		Fertilized female gametophytes					
Pistil	Pollen	PN fusion phenotype	SN fused	SN unfused	SN fusion defect (%)	P value for 50% SN fusion defect	*n*
*gex1-1*/+	*gex1-1*/+	fused	54	7	11.5	1.77 × 10^–9^*	61
		unfused	34	32	48.5	0.806	66
*gex1-1*/+	+/+	fused	55	7	11.3	1.09 × 10^–9^*	62
		unfused	56	7	11.1	6.88 × 10^–10^*	63
+/+	*gex1-1*/+	fused	105	10	8.7	8.09 × 10^–19^*	115
+/+	+/+	fused	91	9	9.0	2.40 × 10^–16^*	100

Pistils of *gex1-1*/+ or wild-type (+/+) plants expressing HISTONE H2B-tdTomato driven by the *RPS5A* promoter were pollinated with pollen from *gex1-1*/+ or wild-type plants expressing HISTONE H2B-GFP driven by the *RPS5A* promoter. The pistils were fixed 8 h after pollination, and then cleared and analyzed by confocal laser-scanning microscopy (CLSM) for fusion of the polar nuclei (PN) and sperm nuclei (SN). ^1^Validation by χ^2^ test. *Significant difference with *P* < 0.01.

The proportion of sperm nuclear fusion defects was approximately 10% for fertilized female gametophytes of the wild-type plants or female gametophytes of the *gex1-1*/+ plants fertilized with wild-type pollen (Table 1). The presence of punctate GFP signals in these fertilized female gametophytes was probably because the fertilization process is in progress in a significant proportion of ovules at 8 HAP. Fertilized female gametophytes fixed before complete sperm nuclear fusion could be counted as sperm nuclear fusion-defective female gametophytes.

**FIGURE 5:**
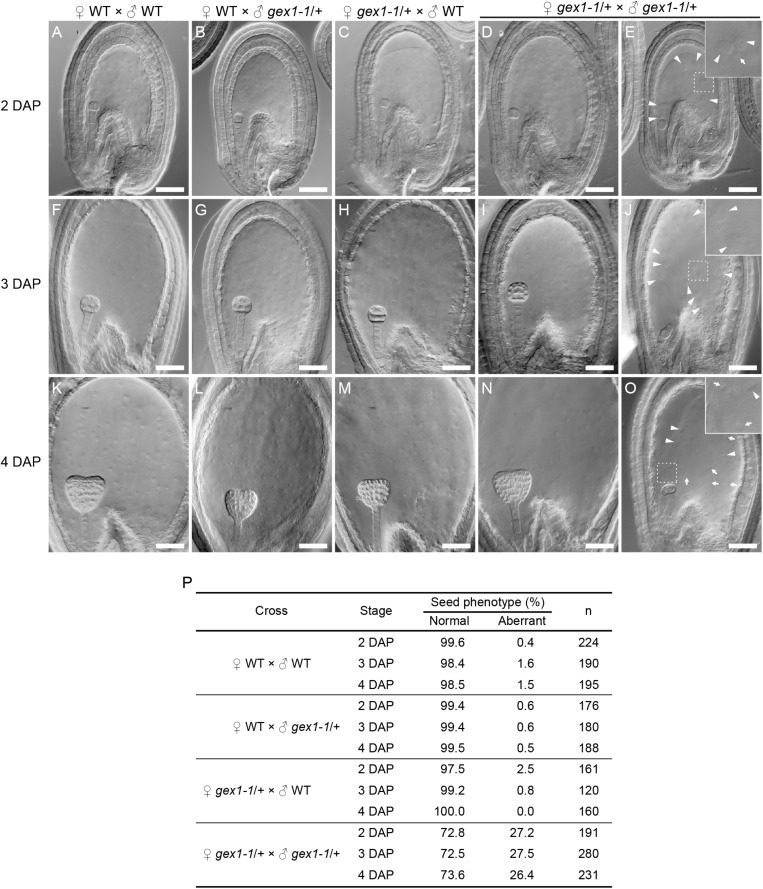
Aberrant seed development after fertilization of a *gex1-1* female gametophyte by *gex1-1* pollen. The pistils of the *gex1-1*/+ or wild-type (WT) plants were pollinated with the pollen from the *gex1-1*/+ or wild-type plants. Seed development in the wild-type pistils pollinated with the pollen from the wild-type **(A,F,K)** or *gex1-1*/+ **(B,G,L)** plants. Seed development in *gex1-1*/+ pistils pollinated with the pollen from the wild-type **(C,H,M)** or *gex1-1*/+ **(D,E,I,J,N,O)** plants. **(A–E)** Seeds 2 days after pollination (DAP). **(F–J)** Seeds 3 DAP. **(K–O)** seeds 4 DAP. In the *gex1-1*/+ pistils pollinated with the pollen from the *gex1-1*/+ plants, three quarter of the seeds developed normally **(D,I,N)**, whereas one quarter of the seeds showed aberrant development in both embryo and endosperm **(E,J,O)**. The arrowheads and arrows in **(E)**, **(J),** and **(O)** indicate the endosperm nuclei that are larger and smaller than those observed in the wild-type seeds, respectively. **(E**,**J,O** Insets) Enlarged images of the framed areas. **(P)** Percentage of normal and aberrant seeds in each cross. Scale bars = 50 μm.

The *gex1-1* homozygous mutant seeds were lethal and showed defects in early embryo development. Analyses of the mutant seeds at 4 days after pollination (DAP) showed embryo developmental arrest between the two- and eight-celled embryos (Alandete-Saez et al., 2011). To investigate whether the sperm nuclear fusion defect caused the seed developmental defect, we analyzed seed development at earlier stages, that is, 2–4 DAP. We pollinated the pistils of the *gex1-1*/+ plants with the pollen of the *gex1-1*/+ plants, fixed, and analyzed seeds after clearing, by differential interference constant imaging. Approximately 27% of the fertilized seeds showed aberrant development, which was not observed when the pistils of the *gex1-1*/+ plants were pollinated with the pollen of the wild-type plants or the pistils of the wild-type plants were pollinated with the pollen of the wild-type or *gex1-1*/+ plants (Figure 5P). The percentage of aberrant seeds found after pollination of the *gex1-1*/+ pistils with the *gex1-1*/+ pollen is consistent with the finding that the *gex1-1*/+ plants showed approximately 25% reduced seed set (Alandete-Saez et al., 2011).

The aberrant seeds showed abnormal endosperm development containing fewer endosperm nuclei, which sometimes showed size irregularities (Figure 5E,J,O; arrows and arrowheads). The abnormal endosperm development was similar to that observed in the *bip1 bip2* and *erdj3a p58^*ipk*^* mutant seeds (Maruyama et al., 2010, 2014b), which showed defects in sperm nuclear fusion in the fertilized central cell (Maruyama et al., 2020). The observed results are consistent with the sperm nuclear fusion defect in the central cell after plasmogamy in the fertilization of the *gex1-1* female gametophyte by *gex1-1* pollen. As reported by Alandete-Saez et al. (2011), the aberrant seeds showed defects in embryo development. At 4 DAP, the aberrant seeds contained an embryo between the two- and eight-celled embryo stages. Embryo development abnormalities were already apparent at 2 DAP. In the wild-type seeds, the embryos were in the two-celled embryo stage at 2 DAP (Figures 5A–D). In contrast, delayed embryo development was observed in the aberrant seeds; 81% and 19% (*n* = 52) contained a one-celled embryo (Figure 5E) and a two-celled embryo, respectively. These results suggest that the first asymmetric cell division occurred after fertilization of the *gex1-1* female gametophyte by *gex1-1* pollen. The one-celled embryo in the aberrant seeds contained a nucleus. Sperm nuclear fusion probably occurred during embryo development.

## Discussion

Nuclear fusion is essential for reproduction. Our results showed that GEX1 is required for nuclear fusion in *Arabidopsis* reproduction. They also showed that mutations in *GEX1* caused defects in nuclear fusion during *Arabidopsis* reproduction. The fusion of polar nuclei in the nuclear membrane fusion step was defective in the *gex1* mutant female gametophytes (Figure 1). Sperm nuclear fusion was also defective in both egg and central cells after plasmogamy in the fertilization of the *gex1-1* mutant female gametophyte by *gex1-1* pollen.

In this study, we observed that half of the mature female gametophytes from the pistils of the *gex1-1*/+ plants contained unfused polar nuclei (Figure 1). This result is in contrast to the findings of Alandete-Saez et al. (2011). The difference is probably because of the differences in the method of analysis of the female gametophytes. Alandete-Saez et al. (2011) used differential interference constant microscopy to observe the ovules and we used CLSM (Christensen et al., 1997) to analyze female gametophyte development. Using CLSM, we were able to quantitatively analyze female gametophytes in a pistil; phenotypes of 94.0% ± 3.0% of the ovules in a pistil were analyzed. The polar nuclear fusion defect of the *gex1-1*/+ mutant was complemented by introducing the *pGEX1:GEX1* construct. We therefore concluded that the *gex1-1* mutant female gametophytes are defective in the fusion of polar nuclei.

While the *gex1-1* mutant allele produced a truncated *GEX1* mRNA encoding a protein lacking the cytoplasmic domain, *gex1-3* is a null *GEX1* allele (Alandete-Saez et al., 2011). The *gex1-3*/+ plants showed defects during male and female gametophyte development. Female gametophyte development was arrested in the functional megaspore stage. GEX1 probably functions in the early stages of female gametophyte development. A truncated GEX1 encoded by *gex1-1* probably had a role in the early stages of female gametophyte development, but failed to support the fusion of polar nuclei.

Using the GFP-GEX1 fusion protein, we showed that GEX1 is a nuclear membrane protein, which is specifically expressed in the egg and central cells of female gametophytes (Figure 2 and Supplementary Figure 2). Consistent with its role in polar nuclear fusion, time-lapse imaging revealed that the GFP-GEX1 signal appeared just before contact of the polar nuclei (Figure 3). The timing of GFP-GEX1 expression differed between the egg and central cells; the GFP-GEX1 signal appeared in the egg cell approximately 80 min later than that in the central cell. This may reflect the differential regulation of gene expression in gametic cells after cellularization. Alternatively, the GFP-GEX1 signal was first detected in the polar nuclei as the polar nuclei were larger in size than the egg nucleus. The detection of the GFP-GEX1 signal in the larger polar nuclei was probably easier. Alandete-Saez et al. (2011) reported that expression driven by the *GEX1* promoter can be detected before cellularization. However, GFP-GEX1 expression was not detected in the four-nucleated stage or after the third nuclear division. As the *GEX1* promoter region used in this study was same as that used by Alandete-Saez et al. (2011), GEX1 expression might also be regulated at the translation level.

Nuclear membrane localization of GFP-GEX1 was also observed in the pollen. Consistent with the findings of Alandete-Saez et al. (2011), we observed the expression of GFP-GEX1 in the vegetative cell, and it was localized to the nuclear membrane. Our results showed nuclear membrane localization of GEX1 in both male and female gametophytes. However, the GFP-GEX1 signal in the sperm cells was very weak, and it was difficult to show the nuclear localization of GFP-GEX1 in the sperm cells. In contrast to Alandete-Saez et al. (2011), Engel et al. (2005) used a longer DNA region as the *GEX1* promoter to show the expression of EGFP in the sperm cells. Engel et al. (2005) also showed that the *GEX1* promoter results in the lower expression level than the *GEX2* promoter and that it shows variable expression. Trascriptomics of *Arabidopsis* sperm cells showed *GEX1* expression in the sperm cells (Borges et al., 2008). A detailed analysis of the *GEX1* promoter region(s) is needed to analyze the localization of GFP-GEX1 in the sperm cell.

In other organisms, the GEX1/Kar5 family proteins localize to the nuclear membrane (Beh et al., 1997; Abrams et al., 2012; Ning et al., 2013). In the present study, we showed that *Arabidopsis* GEX1 is also localized to the nuclear membrane. In contrast, using a transient expression assay, *Arabidopsis* GEX1 was previously reported to localize to the plasma membrane (Engel et al., 2005; Alandete-Saez et al., 2011). In these experiments, C-terminal GFP fusions were used to analyze the subcellular localization of *Arabidopsis* GEX1. GEX1 localization might be altered by C-terminal GFP fusion. Notably, the C-terminal cytoplasmic region of GEX1 is rich in Arg residues (Supplementary Figure 5), which may function as the RXR-type ER retention signal (Zerangue et al., 1999; Ma et al., 2001). We observed dot-like GFP-GEX1 signals in the nuclear membrane, suggesting that GEX1 forms protein complexes. A previous study suggested that GEX1 forms a homodimer through its cytoplasmic domain (Alandete-Saez et al., 2011). The significance of the dot-like GFP-GEX1 signal in the function of GEX1 is unclear. The dot-like signals were observed in the nuclear membrane of the egg cell. This indicates that the GFP-GEX1 dots developed independent of nuclear fusion.

Our results showed that GEX1 is required for sperm nuclear fusion in both fertilized egg and central cells. The homozygous *gex1* mutant was reported to be lethal due to defects in early embryo development, while the endosperm continued to develop (Alandete-Saez et al., 2011). We previously showed that the sperm nuclear fusion defect in the central cell, but not the polar nuclear fusion defect, resulted in aberrant proliferation of the endosperm nucleus (Maruyama et al., 2010; Maruyama et al., 2014b; Maruyama et al., 2020). Nuclear proliferation was not arrested by the deficiency in sperm nuclear fusion in the fertilized central cell, suggesting that fertilization of the central cell caused endosperm proliferation. However, endosperm nuclear proliferation became aberrant due to the fusion of the sperm nucleus during the first nuclear division (Maruyama et al., 2020). Consistent with the sperm nuclear fusion defect in the fertilized central cell, we observed abnormal endosperm proliferation in the homozygous *gex1-1* mutant seeds (Figure 5). In contrast, embryo development was found to arrest shortly after double fertilization in the *gex1-1* homozygous mutant seeds (Figure 5; Alandete-Saez et al., 2011). Although the first asymmetric cell division occurred, there was an apparent delay in cell division at 2 DAP. It is likely that the sperm nuclear fusion defect in the fertilized egg cell affected zygotic cell division. Live imaging of early embryogenesis (Gooh et al., 2015) will help elucidate how the embryo developmental defect was caused by the sperm nuclear fusion defect in the egg cell. Nevertheless, seed development started in the homozygous *gex1-1* mutant seeds after fertilization, which is consistent with the expression of paternally derived genes in the embryo and endosperm (Alandete-Saez et al., 2011).

Our results indicate that GEX1 is a functional ortholog of yeast Kar5. In addition to possessing the conserved CRD within the N-terminal domain, both proteins are likely involved in the same step of nuclear membrane fusion. An electron microscopy analysis has suggested that Kar5 is required after outer nuclear membrane fusion in yeast (Rogers and Rose, 2015). Our results also suggest the roles of GEX1 in the fusion of polar nuclei following outer nuclear membrane fusion (Figures 1G,H). The orthologs of GEX1/Kar5 have been identified in zebrafish, *Chlamydomonas*, and *Plasmodium*, and their roles in nuclear fusion have been reported (Abrams et al., 2012; Ning et al., 2013). All proteins localize to the nuclear membrane and function in nuclear fusion during the reproductive phase. Therefore, the GEX1/Kar5 family proteins, including *Arabidopsis* GEX1, are conserved nuclear membrane proteins required for nuclear membrane fusion during reproduction.

The nuclear fusion machinery functioning in reproduction is conserved between yeast and *Arabidopsis*. BiP and ER-resident J-proteins are required for nuclear membrane fusion in both yeast and *Arabidopsis* (Rose et al., 1989; Ng and Walter, 1996; Nishikawa and Endo, 1997; Brizzio et al., 1999; Nishikawa et al., 2008; Maruyama et al., 2010, 2014b, 2020). The Sad1/UNC84 (SUN) proteins are localized to the inner nuclear membrane and contain a conserved SUN domain at the C-terminus (Zhou et al., 2015). The yeast SUN protein, Mps3/Nep98, is required for nuclear fusion during mating (Nishikawa et al., 2003; Rogers and Rose, 2015). Previously, we showed that the expression of a dominant-negative mutant of SUN in developing *Arabidopsis* female gametophytes resulted in the inhibition of polar nuclear fusion (Hwang et al., 2019). Therefore, the BiP/J-proteins, SUN proteins, and GEX1/Kar5 family proteins probably constitute core factors in nuclear fusion in reproduction. In contrast to the expression of the BiP/J-proteins and SUN proteins in vegetative or somatic tissues, the GEX1/Kar5 family proteins are induced during the reproductive phase. The expression of yeast Kar5 is induced by mating pheromone (Beh et al., 1997). In *Arabidopsis*, GEX1 is expressed in the egg, central, and sperm cells (Figure 2; Engel et al., 2005; Alandete-Saez et al., 2011). Sexual stage-specific expression of GEX1 orthologs has been reported in *Chlamydomonas* and *Plasmodium* (Ning et al., 2013). Therefore, we propose that the GEX1/Kar5 family proteins are key proteins that enable nuclear fusion during the reproductive phase.

**FIGURE 6:**
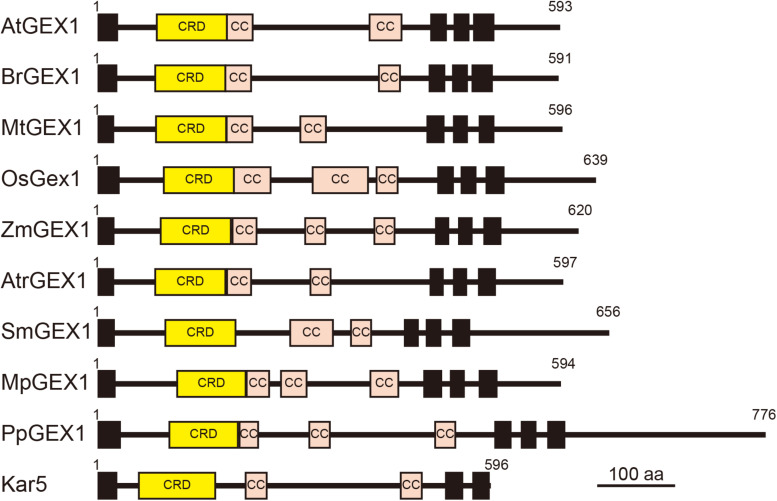
Schematic representation of terrestrial plant orthologs of the GEX1 protein. The GEX1 orthologs of *Arabidopsis* (AtGEX1), *Brassica rapa* (BrGEX1), *Medicago truncatula* (MtGEX1), *Oryza sativa* (OsGEX1), *Zea mays* (ZmGEX1), *Amborella trichopoda* (AtrGEX1), *Selaginella moellendorffii* (SmGEX1), *Marchantia polymorpha* (MpGEX1), and *Physcomitrium patens* (PpGEX1) as well as the yeast Kar5 protein are shown. GEX1 has 1 predicted N-terminal signal sequence (SS), 1 Cys-rich domain (CRD), 2 coiled-coil domains (CC), and 3 transmembrane domains (TMs).

The orthologs of GEX1 have been identified in various land plants, including eudicots, monocots, basal angiosperms, lycophytes, and bryophytes (Engel et al., 2005; Alandete-Saez et al., 2011). All land plant GEX1 orthologs contain the CRD in their N-terminal region followed by two or three putative coiled-coil regions and three transmembrane domains (Figure 6). The CRD is characterized by the presence of six conserved Cys residues (Supplementary Figure 6A). Although several amino acid residues are also conserved between terrestrial plant GEX1 orthologs, sequence similarities between the CRD of terrestrial plant GEX1 orthologs are not high; amino acid identities between eudicot and monocot GEX1 orthologs are approximately 50% (Supplementary Figure 6B). Sequence similarities between terrestrial plant GEX1 orthologs are lower outside the CRD with variations in the putative coiled-coil regions (Figure 6). In contrast to *Arabidopsis*, many angiosperms including wheat and rice produce female gametophytes containing unfused or partially fused polar nuclei in the central cell (You and Jensen, 1985; Jones and Rost, 1989). In maize, mature female gametophytes contain either unfused polar nuclei or a secondary nucleus in the central cell (Mòl et al., 1994). It would be of interest to determine whether such a variation in polar nuclear fusion between species is related to variations in the expression or activity of Gex1. Molecular genetics have enabled the identification of factors required for female gametophyte development in *Arabidopsis* and other species (Erbasol Serbes et al., 2019). We now await the results of comparative analyses of female gametogenesis at the molecular level.

## Data Availability Statement

The raw data supporting the conclusions of this article will be made available by the authors, without undue reservation.

## Author Contributions

SN designed the study. SN, YY, CS, AY, YuS, DK, YoS, DS, TH, and DM performed the experiments. SN and DM prepared the manuscript. All authors contributed to the article and approved the submitted version.

## Conflict of Interest

The authors declare that the research was conducted in the absence of any commercial or financial relationships that could be construed as a potential conflict of interest.
