# Supplementary material for: Arabidopsis GEX1 Is a Nuclear Membrane Protein of Gametes Required for Nuclear Fusion During Reproduction
Source: Front Plant Sci. 2020 Oct 12;11:548032. doi: 10.3389/fpls.2020.548032 (PMC7586128; doi:10.3389/fpls.2020.548032)
Supplement: Supplementary file 3 [file Data_Sheet_1.pdf]

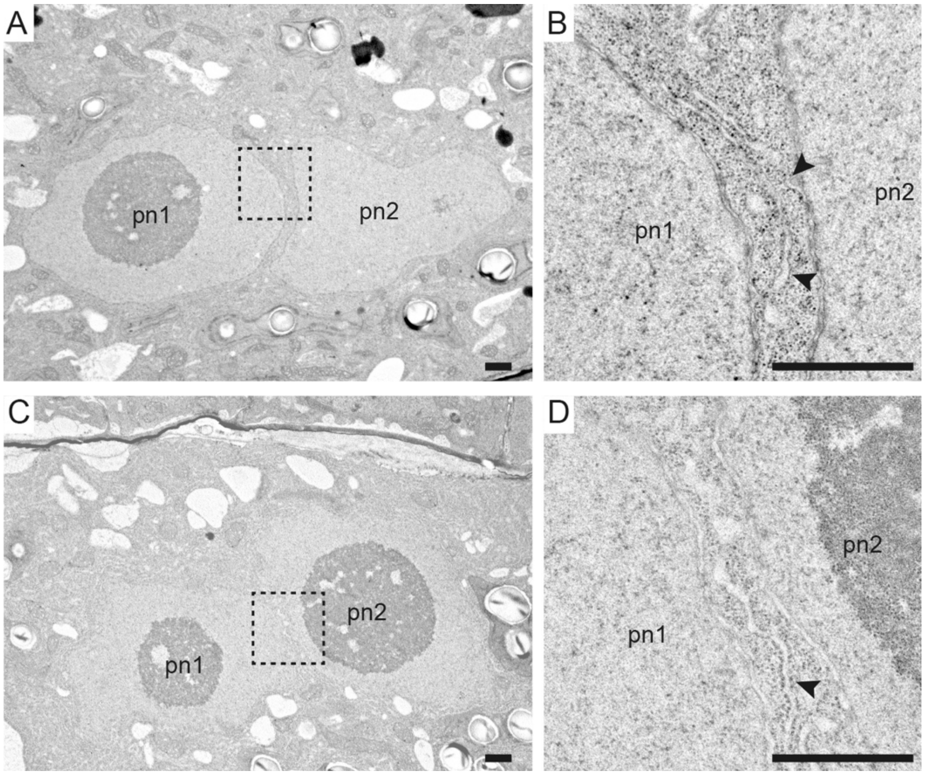

Supplementary Figure 1. Electron micrographs of the central cell of a *gex1-1* female gametophyte containing unfused polar nuclei. Images of two independent female gametophytes are shown. The region indicated with a box in **(A)** and **(C)** is magnified in **(B)** and **(D)**, respectively. The arrowheads show membrane bridges connecting the outer nuclear membrane and the endoplasmic reticulum (ER) membrane. pn1, polar nucleus 1; pn2, polar nucleus 2. Bars = 1 μm

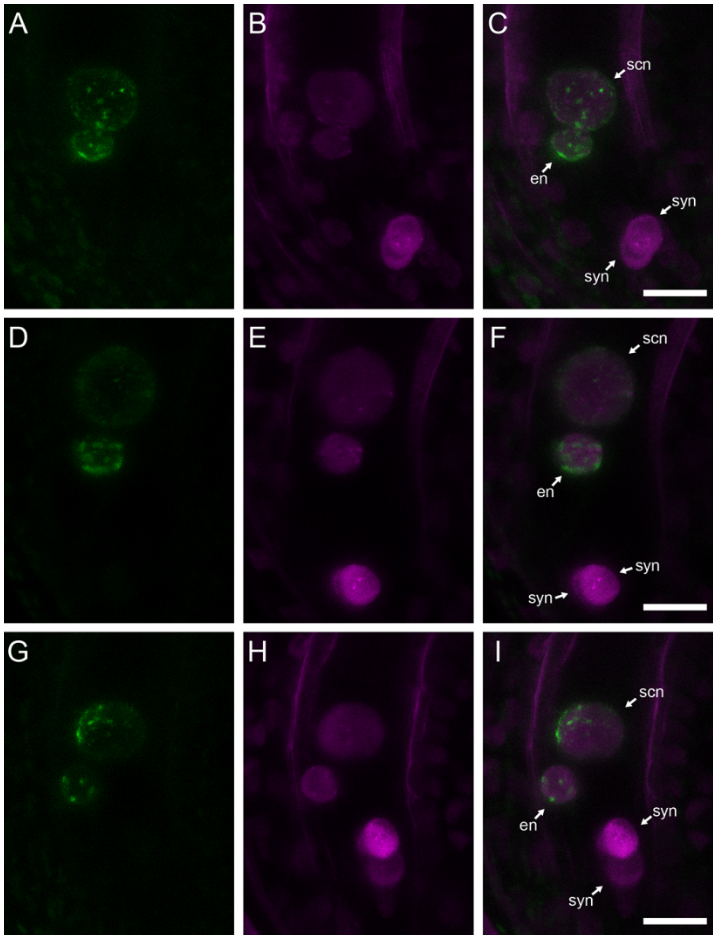

Supplementary Figure 2. CLSM images of a mature wild-type ovule expressing GFP-GEX1 driven by the *GEX1* promoter and HISTONE H2B-tdTomato driven by the *RPS5A* promoter. Magnified images of the egg apparatus regions of three ovules are shown. GFP fluorescence **(A, D, G)**, tdTomato fluorescence **(B, E, H)**, and merged **(C, F, I)** images are shown. syn, synergid nucleus; en, egg nucleus; scn, secondary nucleus. Bars = 10 μm.

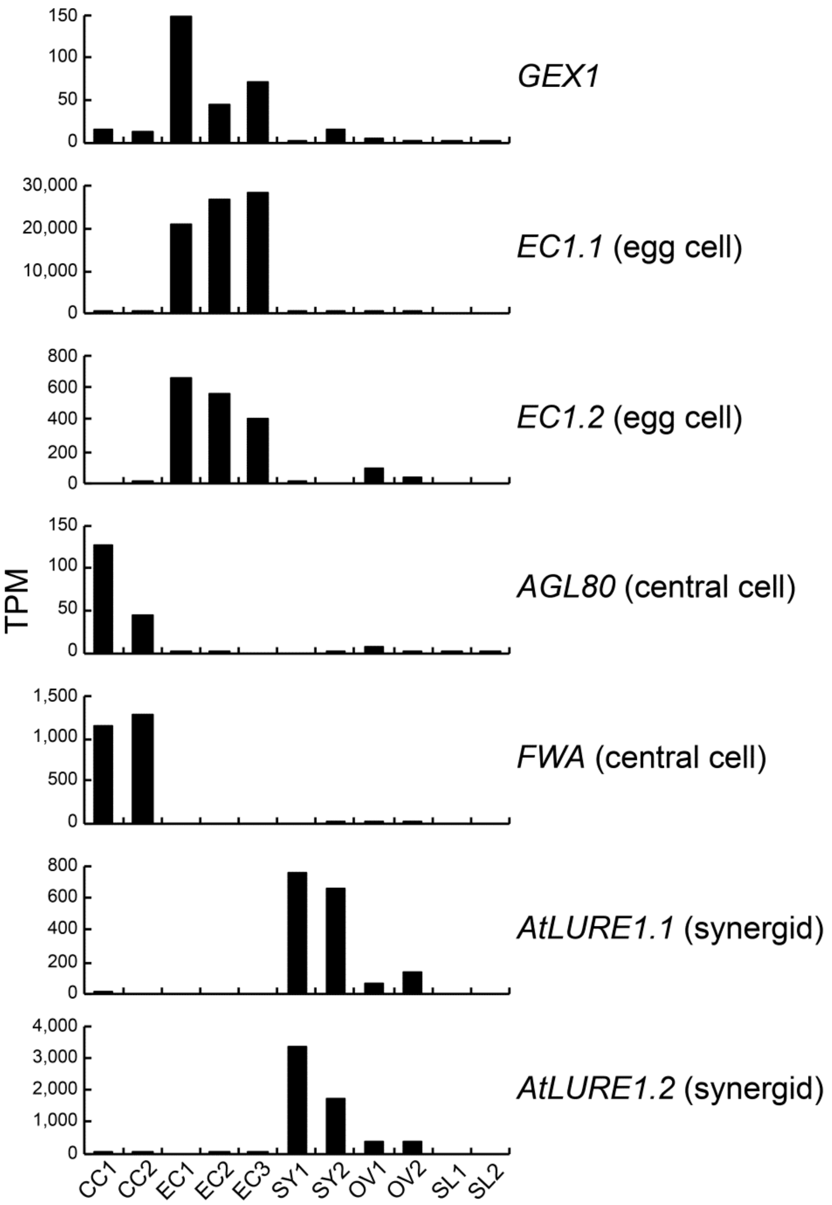

Supplementary Figure 3. Detection of *GEX1* transcripts in the egg and central cells. Transcriptome analysis of the female gametophyte cells is described by Susaki et al. (2020). The biological replicates were analyzed by RNA-Seq for the 2 central cell (CC), 2 egg cell (EC), 3 synergid (SY), 2 ovule (OV), and 2 seedling (SL) samples. The expression of *GEX1* and female gametophyte cell type-specific genes are shown by transcripts per million (TPM) values.

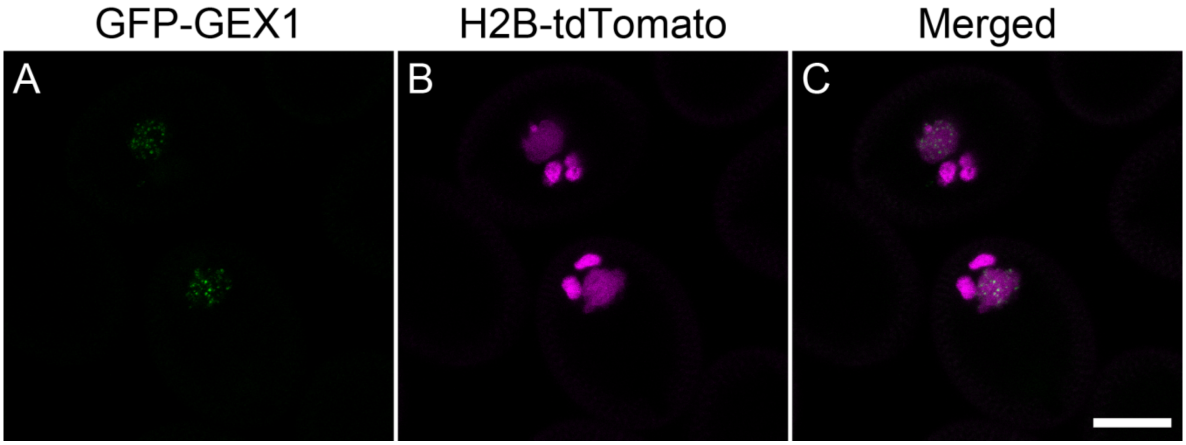

Supplementary Figure 4. CLSM images of wild-type pollens expressing GFP-GEX1 driven by the *GEX1* promoter and HISTONE H2B-tdTomato driven by the *RPS5A* promoter. GFP fluorescence **(A)**, tdTomato fluorescence **(B)**, and merged **(C)** images are shown. Scale bar = 10 μm.

```
AtGEX1_At5g55490     509 RDYEVLNHQILLRLVDKVNDMQSK-----KELSYD---EDTESEVDWTSWVDTDLTDDDD 560
OsGEX1_XP_015611623  522 RDYELLNHHLLQTLVEKVRALEETAAAGEKMLPYGGGGAESERSLMDYSWVFDELADEVD 581
ZmGEX1_NP_001168240  518 RDYEVLNYGLLQTLVEKVRALEENAGGRAP--SY---ASESEESLRDYSWVFDELADDVD 572
                         ****:**: :*  **:**. ::..         *     ::* .:   ***  :*:*: *

AtGEX1_At5g55490     561 NLADPDYKIPLLIKDNPV-------------------TTSSLTRRLYNFRPR------   593
OsGEX1_XP_015611623  582 SNADPSYALPGDEQRQVAVVAPRRRHCASPEEVVGENSITTSAGRRYNLRPRSSYRQT   639
ZmGEX1_NP_001168240  573 SKMDPTYVVPPERSPP-----TRRRDQIVVAEETGENSVTTFVSRKYNLRARK-----   621
                         .  ** * :*   .                       : :: . * **:* *
```

Supplementary Figure 5. Alignment of the C-terminal ends of *Arabidopsis*, rice, and maize GEX1 proteins

The C-terminal of *Arabidopsis* (AtGEX1), rice (OsGEX1), and maize (ZmGEX1) GEX1 orthologs were aligned using Clustal Omega (Sievers et al., 2011). The Arg residues are highlighted.

A

```
AtGEX1_At5g55490       76 LTSCWQNAYSYLLAGCKETIATEEKRKRFAWYLSDCFIKDSGRPAFPTCKDESVMMSCLK 135
BrGEX1_XP_009120059    74 PNSCWQNAYGYLLSGCKGMVATEEQRKRFAWHLSDCFQKESGRPDFPTCNDKQTMMSCLK 133
MtGEX1_XP_013464178    75 SNTCWQNAYQHLFAGCSEILAADEKRSRLAWHLSDCFQRDSGRVSFPRCDAETSIATCLR 134
OsGEX1_XP_015611623    85 PSNCWQEAYRRLFASCGDIMADKEMQSRLAWHLSSCFQEDSGRPPFPRCGEVSDMVHCRK 144
ZmGEX1_NP_001168240    81 PRNCWQDAYGKLFASCGEIMADKERQSRLAWHLSSCFQEDSGRSPFPSCAEGSEMVHCRK 140
AtrGEX1_XP_011625339   74 SNSCWQNAYKALFSSCREIISDQEKKSRLAWHLSDCFHKDSGRSPFPYCDLKSPMSKCLK 133
SmGEX1_XP_024518725    87 QSSCWHSAYSSLFSSCREVLKDDSKKMRLAYKFADCFLRSSGKDPLKSCPDSSPVKECTK 136
MpGEX1_PTQ39308       102 EHSCWQLAYSGMFKSCRDILKDEDRKSRLALRLTDCFLKTSGRCGIKKCADSAPVTKCVK 161
PpGEX1_XP_024381903    91 KHSCWHNAYSDLLSSCREILKEEEKKARLAMRLTNCFLKVSERDAI-HCPDSVPISKCTS 149
                            .**: **  :: .*   :  .. : *:*  ::.** . * :  :  *     :  *

AtGEX1_At5g55490      136 KLDDHEHKIYLDFLLETNTICQQLQSNAFKN 166
BrGEX1_XP_009120059   134 KLDDHEHKIYLEFMLETNTICQQLQSHALKN 164
MtGEX1_XP_013464178   135 NLDDLAHKVYLEFYLETNSICYQLQTHAFKH 165
OsGEX1_XP_015611623   145 RLGVSEDQVFLEFFLETNTLCHQLQAEAFKH 175
ZmGEX1_NP_001168240   141 RLGESQDKVFLEFFLETNTLCHQLQAEAFKH 171
AtrGEX1_XP_011625339  134 NIDEGAHKVYLEFFLEVNSICHHLQTDAFKH 164
SmGEX1_XP_024518725   137 SLTDHRHHLLLQFFIDIASMCHHLQSEAFKL 177
MpGEX1_PTQ39308       162 ELDDHTHAIFLAFFIDAASMCHYLQSQEFKL 190
PpGEX1_XP_024381903   150 GLSDHINSIFLAFFIDAASMCHHLQSEAFKQ 180
                           :    . : * * ::  ::*  **:. :*
```

B

	BrGEX1	MtGEX1	OsGEX1	ZmGEX1	AtrGEX1	SmGEX1	MpGEX1	PpGEX1
AtGEX1	78.0	58.2	49.4	49.4	55.0	38.5	40.7	43.3
BrGEX1		57.1	49.5	49.5	53.9	34.1	38.5	38.9
MtGEX1			53.9	55.0	64.9	37.4	38.5	35.6
OsGEX1				84.6	55.0	37.4	38.5	35.6
ZmGEX1					59.3	38.5	39.6	36.7
AtrGEX1						46.2	46.2	46.6
SmGEX1							56.0	58.9
MpGEX1								62.2

Supplementary Figure 6. Alignment of the cys-rich domain (CRD) of terrestrial plant GEX1 orthologs

(A) CRD of *Arabidopsis* (AtGEX1), *Brassica rapa* (BrGex1), *Medicago truncatula* (MtGEX1*), Oryza sativa* (OsGEX1), *Zea mays* (ZmGEX1), *Amborella trichopoda* (AtrGEX1), *Selaginella moellendorffii* (SmGEX1), *Marchantia polymorpha* (MpGEX1), and *Physcomitrium patens* (PpGEX1) GEX1 orthologs were aligned using Clustal Omega (Sievers et al., 2011). The conserved Cys residues in the CRD are highlighted. **(B)** Amino acid identities (%) between the CRD of terrestrial plant GEX1 orthologs.

Supplementary Table 1. List of primers used in this study.

Primer name	Primer sequence (5'–3')
GEX1-YY2	CTATCGTGGACGGAAATTATACAA
GEX1-YY5	CACCATGGATCGTTTCAGCAGAAAATGT
GFPGEX1-1	GCCGCCCCCTTCACCATGGATCGTTTCAGCAGAAA
GFPGEX1-2	CCAACTGTGGCATGTTAATGG
GFPGEX1-3	ACATGCCACAGTTGGATGGTGAGCAAGGGCGAGGA
GFPGEX1-4	ACCATCCCTTGTACAGCTCGTCCATGCC
GFPGEX1-5	TGTACAAGGGATGGTTCTCTTCTTCTTCT
GFPGEX1-6	GGCGCGCCCACCCTTCTATCGTGGACGGAAATTAT
GEX1proF	GGCCAGTGCCAAGCTGCTTAAGGAAGTCAACTCTCTTTGT
GEX1proR	GCAGGCATGCAAGCTTTAATCGGATTTGAGATCTTCTTCT

Supplementary Table 2. Complementation of the polar nuclear fusion defect by the *GEX1* transgene

Mutation	Transgene (homozygous)	Line no.	Polar nuclei unfused (%)	Polar nuclei fused (%)	Total ovules
gex1-1/+	*pGEX1*::*GEX1*	1	3	97	103
		2	8	92	153
		3	3	97	124
		4	1	99	139
		5	6	94	236

The ovules were analyzed by confocal laser-scanning microscopy.

Supplementary Movie 1. Time-lapse imaging of GFP-GEX1 in the developing female gametophyte.

Ovules of a transgenic plant expressing GFP-GEX1 driven by the *GEX1* promoter and HISTONE H2B-tdTomato driven by the *RPS5A* promoter were dissected from the pistils of stage 12 flowers and analyzed by confocal laser-scanning microscopy. Images were captured at 5-min intervals. Two movies of independent ovules (#1 and #2) are shown. Time (h:min) from the metaphase of the third mitotic division is shown. Scale bar = 25 μm

Supplementary Movie 2. Time-lapse imaging of GFP-GEX1 during the poplar nuclear fusion process.

Ovules of a transgenic plant expressing GFP-GEX1 driven by the *GEX1* promoter and HISTONE H2B-tdTomato driven by the *RPS5A* promoter were dissected from pistils of stage 12 flowers and analyzed by confocal laser-scanning microscopy. Images were captured at 5-min intervals. Two movies of independent ovules (#1 and #2) are shown. Time (h:min) from the contact of two polar nuclei is shown. Scale bar = 25 μm

REFERENCE

Sievers, F., Wilm, A., Dineen, D., Gibson, T. J., Karplus, K., Li, W., Lopez, R., McWilliam, H., Remmert, M., Söding, J., Thompson, J. D., and Higgins, D. G. (2011). Fast, scalable generation of high-quality protein multiple sequence alignments using Clustal Omega. *Mol. Syst. Biol.* 7, 539. doi: 10.1038/msb.2011.75
